# A Review Paper on Optical Coherence Tomography Evaluation of Coronary Calcification Pattern: Is It Relevant Today?

**DOI:** 10.3390/jcdd11080231

**Published:** 2024-07-24

**Authors:** Horea-Laurentiu Onea, Maria Olinic, Florin-Leontin Lazar, Calin Homorodean, Mihai Claudiu Ober, Mihail Spinu, Alexandru Achim, Dan Alexandru Tataru, Dan Mircea Olinic

**Affiliations:** 1 Department of Internal Medicine, Medical Clinic Number 1, “Iuliu Hatieganu” University of Medicine and Pharmacy, 400347 Cluj-Napoca, Romania; onea.lau@gmail.com (H.-L.O.); leontinlazar@gmail.com (F.-L.L.); calinhomorodean@gmail.com (C.H.); spinumihail04@gmail.com (M.S.); tataru.cardio@gmail.com (D.A.T.); danolinic@gmail.com (D.M.O.); 2 County Clinical Emergency Hospital Sibiu, 550024 Sibiu, Romania; 3 Second Cardiology Department, County Clinical Emergency Hospital Cluj-Napoca, 400347 Cluj-Napoca, Romania; mober2009@gmail.com; 4 Niculae Stancioiu Heart Institute Cluj-Napoca, 400001 Cluj-Napoca, Romania; dr.alex.achim@gmail.com

**Keywords:** optical coherence tomography, coronary artery disease, vulnerable plaque, calcification pattern, spotty calcification, calcified nodule, calcified protrusion, superficial calcified plate

## Abstract

The process of coronary calcification represents one of the numerous pathophysiological mechanisms involved in the atherosclerosis continuum. Optical coherence tomography (OCT) represents an ideal imaging modality to assess plaque components, especially calcium. Different calcification patterns have been contemporarily described in both early stages and advanced atherosclerosis. Microcalcifications and spotty calcifications correlate positively with macrophage burden and inflammatory markers and are more frequently found in the superficial layers of ruptured plaques in acute coronary syndrome patients. More compact, extensive calcification may reflect a later stage of the disease and was traditionally associated with plaque stability. Nevertheless, a small number of culprit coronary lesions demonstrates the presence of dense calcified plaques. The purpose of the current paper is to review the most recent OCT data on coronary calcification and the interrelation between calcification pattern and plaque vulnerability. How different calcified plaques influence treatment strategies and associated prognostic implications is of great interest.

## 1. Introduction

Optical coherence tomography (OCT), a light-based intravascular imaging modality, offers high-resolution cross-sectional images of the vessel wall (10–20 µm) and has emerged as an important adjuvant to basic coronary angiography [1]. Through optimal tissue characterization, it can help guide percutaneous coronary interventions (PCI) by allowing selection of the appropriate treatment strategy and it can identify the culprit plaque as well as the mechanism of subsequent plaque destabilization. Equally significant, OCT demonstrates its utility in both intra- and post-PCI settings by facilitating proper stent sizing, guidance of complex scenarios (left main disease or coronary bifurcations), stent optimization, and identification of stent-related complications [2,3,4]. As compared to intravascular ultrasound (IVUS), it allows far better visualization and characterization of calcific components at the cost of lower penetration capability [1].

Coronary artery calcification represents a dynamic process found in both the early and late stages of atherosclerosis with significant impact on the management of coronary artery disease (CAD). As seen on pathology [5], the progression of calcification typically starts with microcalcifications (MC) which grow into larger fragments forming spotty calcifications (SC) and eventually diffuse sheet-like deposits. Sheet calcifications may fracture, leading to the formation of nodular calcifications, which may protrude into the lumen or media. While the extent of coronary calcification quantified through the coronary artery calcium scoring has traditionally been considered a predictor of adverse clinic events [6], the effect of calcification on plaque stability is still under debate. Previous histology [7] and ^18^F-fluoride positron emission tomography [8] studies have suggested that microcalcifications are associated with plaque rupture in high-risk plaques. Spotty calcifications are associated with more diffuse atherosclerosis and accelerated disease progression [9] and are more frequently located in culprit plaques of acute coronary syndrome (ACS) patients [10].

More compact, extensive calcification may reflect a later stage of atherosclerosis and was commonly thought to promote plaque stability [11]. Nevertheless, more recently, the presence of dense calcium was observed at the culprit lesions of ACS patients. Three distinct entities were described, according to OCT imaging [12]: superficial calcified plates (SCP), eruptive calcified nodules (CN), and calcified protrusions (CP), respectively. Whereas CN is an established mechanism of plaque destabilization found in up to 8% of ACS cases [13], data on SCP and CP are still limited. This OCT review will focus mainly on the implications of calcification pattern on plaque vulnerability, how it influences treatment and management, and patient prognosis. A PubMed search was conducted using the following key words: optical coherence tomography; coronary artery disease; vulnerable plaque; calcification pattern; spotty calcification; calcified nodule; calcified protrusion; superficial calcified plate.

## 2. Microcalcification

Early coronary calcification process with the development of MC seems to originate from both macrophage and smooth muscle cell apoptosis and release of matrix vesicles [5]. A distinction must be made between cellular-level MC versus OCT-defined MC. Calcifications ranging from 0.5 to 15 µm in size fall below the resolution of current OCT devices, and what is more, even when noticeable can be confounded with other entities that present as OCT bright spots [14]. Milzi et al. [15] proposed a practical classification of calcification size based on OCT-derived maximum calcium arc and length.

Important insights into the pathophysiology of ACS come from papers published by Virmani et al. [16] and later by Vengrenyuk et al. [17,18]. The former showed that a thin-cap fibroatheroma (TCFA), defined as a large necrotic core encapsulated by a thin (cutoff 65 µm) fibrous cap heavily infiltrated by macrophages, represents a precursor to plaque destabilization and subsequent plaque rupture. The latter demonstrated the existence of MC in fibrous caps, which can increase local stress by a factor of 2 independent of their relative location in the cap. These cellular-level MC by themselves do not seem to be detrimental unless they are located in an area of increased background stress, such as a thin fibrous cap. Subsequently, at a cap thickness of <65 µm, a calcified macrophage located in an area of increased stress (>300 kPa) can intensify this stress to ~600 kPa, thus providing an explanation to the observation that not all plaque ruptures occur near the areas of maximum stress. Interestingly, calcification shape is relevant, as elongated MC seem to be more dangerous than spherical MC. With the help of a high-resolution (2.1 µm) microcomputed tomography capable of detecting structures as small as 5 µm it has been revealed that MC are not that uncommon and almost all fibrous caps contain MC (vast majority <15 µm) [19]. While particles <5 µm seem to be nonharmful, those >5 µm consist of conglomerates of smaller ones, which—if located in close enough proximity—are capable of generating a much greater stress factor compared to single MC.

**Figure 1:**
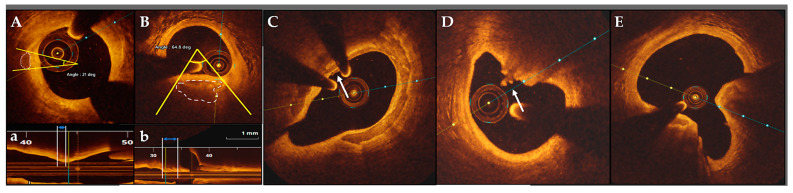
Representative images of each OCT-identified calcification pattern. (**A**) Microcalcification with calcium arc < 22.5° and (**a**) calcium length < 1 mm. (**B**) Spotty calcification with calcium arc < 90° and (**b**) calcium length < 4 mm. (**C**) Superficial calcified plates with evidence of a small white thrombus (white arrow). (**D**) Eruptive calcified nodule, white arrow indicating expulsion of small nodules. (**E**) Calcified protrusion. OCT: optical coherence tomography.

According to OCT imaging, MC are identified when the maximum calcium angle is <22.5° and calcification length is <1 mm [15] (Figure 1). In a study including stable CAD patients MC were present in 18.7% of target lesions [20]. In plaques containing MC, there was a higher number of calcifications per lesion, a lower percentage of area stenosis, and a higher frequency of macrophage infiltration as well as calcium–macrophage co-localization, as compared to those without (6.7 ± 3.0 vs. 3.2 ± 2.5, *p* < 0.001; 70.9 ± 11.1 vs. 76.2 ± 9.7%, *p* = 0.028; 66.7 vs. 37.4%, *p* = 0.014; 47.6 vs. 15.6%, *p* = 0.001). At the multivariate analysis, only the total number of calcifications per lesion (OR 1.53, 95% CI 1.23–1.91, *p* < 0.001) and average macrophage angle (OR 1.28 for 10°-variation, 95% CI 1.03–1.60, *p* = 0.024) were independent predictors for the presence of MC. This association between a less severe coronary stenosis and co-localization of plaque vulnerability features reinforces the idea that MC represent an early stage in the atherosclerosis process. Further expanding the concept, Burgmaier et al. [21] prospectively enrolled 155 patients, most of them with stable CAD (75%), and investigated the predictive role of OCT-detected calcium–macrophage co-localization in terms of cardiovascular (CV) outcome. At a median follow-up of 5.4 years, patients with calcium–macrophage co-localization show a significant higher incidence of the composite endpoint (death from any cause, myocardial infarction—MI and coronary revascularization) even after adjustment for CV risk factors (HR 1.83, 95% CI 1.03–3.24, *p* = 0.039; (HR 1.98, 95% CI 1.07–3.67, *p* = 0.030). It is noteworthy that the presence of MC was associated with larger necrotic cores in an ACS setting [22]. With diabetes mellitus (DM) being a known risk factor for CAD severity and progression, Milzi et al. conducted a retrospective study comparing plaque morphology using OCT in DM vs. non-DM patients [15]. The study excluded patients presenting with ACS. A similar amount of MC within the target vessel were detected between the two groups (0.34 ± 0.79 vs. 0.31 ± 0.71), which also applied for the number of spotty, macro, and superficial calcifications.

A recent pilot study integrating OCT data with computational fluid dynamics and 18F-sodium fluoride positron emission tomography investigated the relation between endothelial shear stress and high-risk plaque features (including MC) in ACS [23]. Low endothelial shear stress, a known predictor of TCFA thinning [24], was found to correlate positively with MC and inflammation both at the segment (r_s_ = 0.52; *p* = 0.001; r_s_ = 0.33; *p* = 0.043) and artery level (r_s_ = 0.64; *p* = 0.002; r_s_ = 0.46; *p* = 0.041). Integrating shear stress calculation into imaging assessment may provide additional useful information, thus allowing improved management of CAD in the future.

## 3. Spotty Calcification

SC are hypothesized to originate from progressive fusion of smaller MC over time in a centrifugal pattern from the outer rim of the necrotic core to the adjacent collagenous matrix [25] and are identified on OCT in the presence of a lesion between 1 to 4 mm in length with an arc of calcification <90° [15] (Figure 1). Burke et al. [26] speculate that SC can also be promoted by asymptomatic plaque rupture and healing.

In a stable CAD cohort of 300 patients [27], non-culprit lipid-rich plaques within the target vessel requiring PCI were examined using OCT imaging. SC were prevalent in 39.6% of plaques, which showed a higher lipid burden, thinner fibrous caps (89.0 ± 31.6 vs. 136.5 ± 32.5 µm, *p* = 0.002) and a higher microchannel (45.9 vs. 17.7%, *p* = 0.007) and macrophage content (47.7 vs. 20.2%, *p* < 0.0001) as compared to SC-free plaques. A positive correlation was found between the number of SC and fibrous cap thickness (r = −0.40, *p* = 0.006), as well as the prevalence of microchannels (*p* = 0.01). The presence of SC is associated with other vulnerability features (macrophage content—*p* = 0.001; cholesterol crystals—*p* < 0.001) in an acute setting as well [28]. Mizukoshi et al. [29] evaluated calcium deposits at the culprit lesion in patients with ACS and stable CAD. The number of SC was significantly higher, while the calcium burden (arc, area, and length) and number of large calcifications were significantly lower in the ACS group as compared to the stable group (all *p* < 0.001). SC were more superficial in the ACS group as compared to the stable group (60–70 vs. 193 µm, *p* < 0.001), while an increase in their number can increase plaque vulnerability and risk of rupture (r = 0.479, *p* < 0.001). In a prospective study including 98 consecutive ACS patients [30], both the total number (79 vs. 50%, *p* = 0.006) as well as the number of SC per patient (1.5 ± 1.0 vs. 0.8 ± 0.9, *p* < 0.001) were significantly higher in ruptured plaques as compared to non-ruptured plaques. Moreover, in the rupture group, most calcifications tended to be shallower, located near the plaque rupture site (70% within 4 mm proximal and 2 mm distal), and interestingly, they were proximal relative to the minimum lumen area (MLA) site (70% within 4 mm). The latter finding comes in concordance with previous studies demonstrating that the maximum necrotic core area was located proximal to the MLA site (66 vs. 5%) and was associated with a higher frequency of TCFA (24 vs. 9%, *p* < 0.001) [31].

The role of SC in plaque destabilization was recently disputed in a paper by Ong et al. [32]. The team found no differences in the number of SC (*p* = 0.87) or large calcifications (*p* = 0.27) when comparing ST-elevation myocardial infarction (STEMI) with stable CAD patients. A plausible explanation could reside in different methodology compared to previous studies, by focusing the OCT analysis on a 10 mm culprit segment vs. a longer 30 mm segment [29]. Considering that ruptured culprit plaques measure on average 16.1 mm in length [33], including more distant calcium deposits may be superfluous as they are unlikely to increase the focal biomechanical stress.

Data on the prognostic impact of SC on cardiovascular outcomes is to date very limited. The OPTICO-ACS study [34] prospectively enrolled 155 consecutive ACS patients investigated by means of OCT imaging and simultaneous immunophenotyping using flow-cytometric analysis and the cytokine bead array technique. At the 12-month follow-up, the major adverse cardiovascular event (MACE) rate was higher in patients exhibiting SC as compared to those without (16. vs. 5.3%, *p* < 0.05). Furthermore, a specific inflammatory profile was identified in the former group, characterized by increased circulating levels of neutrophils (0.96 (0.85) vs. 0.91 (0.77); *p* < 0.05), TNF-α (1.17 (0.93) vs. 1.06 (0.89); *p* < 0.05), IL-8 (2.04 (1.24) vs. 1.37 (1.10); *p* < 0.05) and intermediate monocytes expressing CD49d receptors (1.06 (0.94) vs. 0.97 (0.91); *p* < 0.05). Future therapeutical directions in the management of ACS could be tailored to tackle these pathopsychological pathways. In a smaller OCT study including stable patients [35] the colocalization of TCFA and SC was a powerful predictor of PCI-related MI (OR 21.00, 95% CI 2.65–454.22, *p* = 0.003) while another IVUS study [36] found deep SC (defined as located within the deeper 50% of the plaque) as an independent predictor of non-culprit lesion-related revascularization during 6 years of follow-up (OR 5.93; 95% CI 3.08–154, *p* = 0.002). The latter finding suggests that deep SC may contribute to chronic plaque progression and need for future revascularization. Pu et al. [37] conducted a prospective study of cardiac arrest survivors undergoing immediate or delayed revascularization, who underwent intravascular imaging (OCT or IVUS). The team found that SP (especially superficial) was an independent predictor for post-PCI no-reflow phenomenon (OR 3.09, 95% CI 1.43–21.77, *p* = 0.021).

Intracoronary imaging screening for the presence of SC may be useful as it can guide optimal medical therapy. A post hoc analysis [38] of a randomized controlled trial compared the effect of moderate vs. intensive statin therapy on 96 non-culprit lipid-rich plaques in terms of plaque characteristics using OCT. Unsurprisingly, compared to baseline, at the 6- and 12-month follow-ups there was an increase in fibrous cap thickness in both the SC as well as the non-calcified group (62.8 ± 20.9, 126.4 ± 84.9, and 169.2 ± 81.6 μm, *p* < 0.001; 60.0 ± 17.2, 125.5 ± 62.1, and 161.0 ± 80.5 μm, *p* < 0.001). Furthermore, in plaques containing SC, intensive statin therapy was more effective than moderate statin therapy in increasing fibrous cap thickness (*p* = 0.034).

**Figure 2:**
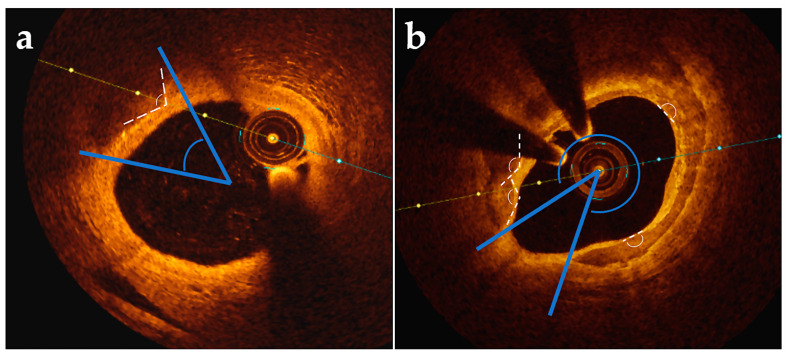
Example of intrinsic calcification angle measurement in comparison to calcium arc (**a**) in case of a spotty calcification (calcium arc < 90°); (**b**) in case of macrocalcification or irregular calcification, the smallest ICA for every OCT section was considered. ICA: intrinsic calcification angle; OCT: optical coherence tomography.

Reith et al. [39] introduce a novel parameter of plaque vulnerability defined as the intrinsic calcification angle (Figure 2), which represents the angle externally projected by a vascular calcification. Mean intrinsic calcification angle was lower in ACS vs. stable patients (164.1 ± 14.3° vs. 176.0 ± 8.4°, *p* < 0.001) and was a good predictor for the occurrence of ACS (AUC = 0.840, 95% CI 0.797–0.882, *p* < 0.001, optimal cut-off 175.9°). Furthermore, in finite elements analysis, lower intrinsic calcification angle increases cap stress, which can additionally increase with more superficial calcifications.

## 4. Macrocalcification

As plaque progresses calcifications become larger, forming diffuse areas of calcification described in histology as sheets of calcium that can incorporate both the collagenous matrix and necrotic core [40]. Macrocalcifications were defined on OCT as calcifications extending on an arc greater than 90° or more than 4 mm in length [15,30,32]. Coronary calcium scoring is a computed tomography surrogate for the extent of coronary artery calcification that has been traditionally used as a predictor of future CV events [41]. This method seems effective in identifying vulnerable patients not plaques, as several studies have shown that the presence of macrocalcifications may stabilize plaques.

In the study by Mizukoshi et al. [29], calcium burden was significantly smaller in the ACS group as compared to the stable group and the number of large calcifications inversely correlated with the frequency of plaque rupture (r = −0.219, *p* = 0.003). Other studies [30,32] found no difference in the frequency of large calcifications between ruptured and non-ruptured plaques. Qin et al. [28] found smaller calcification thickness (*p* = 0.008), arc (*p* = 0.008) and length (*p* = 0.007) in ruptured vs. non-ruptured plaques. The presence of macrocalcifications did not increase the risk of plaque rupture (*p* = 0.468) as compared to plaques without calcifications at the culprit site but was associated with higher macrophage (*p* = 0.001) and cholesterol crystal (*p* < 0.001) contents. Nevertheless, TCFA and fibrous cap thickness were similar between the macrocalcification and non-calcification groups.

A recent paper [42] evaluated by means of serial OCT imaging the factors involved in the progression of coronary calcification. At baseline, macrocalcifications were detected in 40% of cases, while SC were detected in 55% of cases. At discharge, almost all of the patients received statin, and at least 1 received an antiplatelet drug. After 6 months, there was an increase in the calcification index in 95% of calcified plaques (132.0 to 178.2, *p* < 0.001) and a parallel decline in unstable plaque features (TCFA, 21.2 to 11.9%, *p* = 0.003; macrophages, 74.6 to 61.0%, *p* = 0.001). Interestingly, macrocalcification content increased to 47%, while SC decreased to 51%, reaffirming the role of macrocalcifications in plaque stability. DM (OR, 3.911; *p* < 0.001), chronic kidney disease (OR, 2.432; *p* = 0.037) and macrophages (OR, 6.782; *p* < 0.001) were found to be independent predictors for rapid progression of calcification.

The relation between CAD and serum biomarkers has been widely investigated. Chu et al. [43] evaluated 420 CAD patients who had undergone OCT imaging and were divided into 2 groups based on serum uric acid levels (cutoff = 6.0 mg/dL). Plaques in the high uric acid group had longer calcification length (6.77 vs. 4.20 mm, *p* = 0.04), while at correlation analysis there was a strong positive association between the 2 parameters (r = 0.386, *p* = 0.006). Another study assessed the association between serum 1,5-anhydro-D-glucitol and coronary calcification in DM patients [44]. There were significantly lower levels of 1,5-anhydro-D-glucitol in DM vs. non-DM patients (*p* = 0.016) as well as fibrocalcified vs. fibrotic or fibrolipidic lesions (*p* < 0.001). In addition, this biomarker correlated negatively with the calcification index (r = −0.729, *p* < 0.001) and at low levels was found as a predictor for MACE in DM patients (*p* = 0.043).

**Figure 3:**
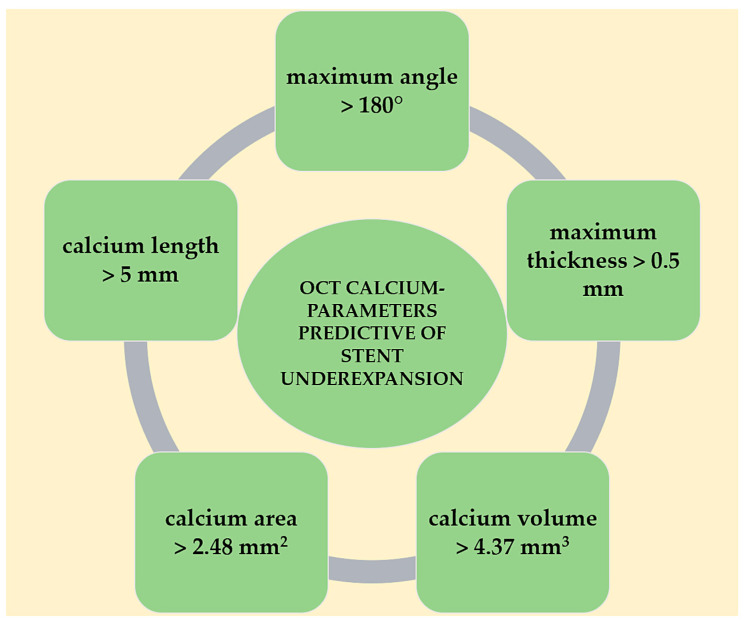
OCT-derived calcium parameters predictive of stent underexpansion. OCT: optical coherence tomography.

A high calcium burden constitutes a challenge in modern-day PCI and, even with the current armamentarium of plaque modification techniques, still leads to high rates of MACE, particularly through target lesion revascularization (TLR) at both short- and long-term follow-up [45]. The mechanisms involved are numerous and include stent underexpansion, stent malapposition, stent fracture, and edge dissection. Stent underexpansion and subsequent small minimum stent area (MSA) remain the most powerful predictors of stent failure in this context [46]. OCT was compared to IVUS in achieving optimal stent expansion during PCI of calcified lesions [47]. Patients were divided into four groups based on the imaging method and use of rotational atherectomy (RA). OCT-guided RA was superior in terms of stent expansion compared to IVUS-guided RA (median 88.0%, interquartile range [78.0–96.0] vs. 76.5% [71.0–84.3], *p* = 0.008), and moreover obtained similar results compared to OCT-guided non-RA. Median minimum calcium thickness was significantly reduced from 800 to 550 µm (*p* < 0.001) using OCT-guided RA. Fujino et al. [48] developed an OCT-calcium-based scoring system to predict which patients would benefit the most from advanced plaque debulking strategies prior to stent implantation. Lesions with a calcium score of 4—consisting of calcium deposits with maximum angle >180°, maximum thickness >0.5 mm, and length >5 mm—are at higher risk of stent underexpansion. The same parameters seem to increase the risk of stent malapposition as compared to lesions with a lower calcium score (84.0% vs. 47.4%, *p* < 0.001), as shown in a recent retrospective study including 336 patients [49]. Furthermore, calcium length was the most robust independent OCT-derived predictor of MSA (MD −0.28 mm^2^/5 mm, *p* = 0.001), while total stent length was predictive of stent expansion (MD −0.465%/mm, *p* = 0.001). More recently, in an attempt to find more accurate OCT parameters to predict stent expansion, Ma et al. [50] identified a moderate correlation between calcium burden, maximum calcium area, calcium volume, and stent expansion. The optimal cut-offs for predicting stent expansion for calcium volume and area were 4.37 mm^3^ and 2.48 mm^2^, respectively (Figure 3).

Suboptimal stent expansion can also result from acute stent recoil due to heavy calcified lesions, which is particularly the case with newer generation thin-strut drug-eluting stents (DES). Chronic stent recoil in this setting was investigated by Amemiya et al. [51] on a small sample of patients (23% on hemodialysis) with good lesion preparation using RA. At the 8-month follow-up chronic stent recoil occurred in 36% of patients, with no significant change in MLA (6.0 to 6.0 mm^2^, *p* = 0.51). Interestingly, within the recoil segments calcium evolved from calcified plaque with a necrotic core to more mature calcium, while the decrease in stent circumference was mainly within non-calcified plaque, probably due to negative remodeling of non-calcified vessel wall.

In a recent study [52] including patients with heavily calcified lesions treated optimally with RA followed by high pressure stent implantation incomplete stent apposition was detected in an overwhelming proportion (98% of cases, mean maximum strut–vessel wall distance = 713 ± 371 μm). Serial OCT images were available in 11 out of the 52 lesions and stent malapposition persisted in 82% of cases, granting there was a significant reduction in max strut-vessel wall distance (692.1 to 462.5 μm, *p* < 0.01). By means of a hybrid imaging technique using virtual histology IVUS-OCT, dense calcium plaque volume was the most robust independent predictor of late stent malapposition [53]. Ueda et al. [54] sought to find the factors associated with late-acquired uncovered stent struts. Large calcification and TCFA were more frequently observed in the poorly covered group compared to the well-covered group (27.6 vs. 9.0%, *p* = 0.017; 10.3 vs. 0.0%, *p* = 0.0032), while at multivariate analysis TCFA and large calcification at the proximal edge, but not the distal edge, were independent predictors of uncovered stent struts.

## 5. Superficial Calcified Plate

Calcium-related risk of plaque vulnerability is dependent not only on calcification shape and size, but its distribution within the vessel wall may also be a significant contributor. Brown et al. [55] demonstrated through biomechanical modelling that plaque structural stress is increased in high-risk non-culprit lesions responsible for MACE, larger and more superficial calcifications being a contributing factor. Moreover, a correlation was demonstrated between thinner and longer calcification, especially when the calcification was more superficial, and an increased stress concentration at the calcium–fibrotic tissue interface [56]. On the contrary, finite element analysis shows that superficial calcifications in close proximity to a lipid pool can lower cap stress [57].

Matsumoto et al. [58] were the first to describe the existence of superficial calcifications on OCT imaging in the form of protruding or non-protruding nodules in a stable patient cohort. More recently, SCP (Figure 1), defined as sheet-like superficial calcium compact deposits without erupted nodules or protruding mass into the lumen [12], were identified and introduced as a novel substrate for plaque destabilization and thrombus formation in patients with ACS [12,59,60]. Table 1 summarizes the main OCT studies on SCP. In a hybrid histology–OCT study, OCT was demonstrated to have an excellent discriminatory capacity for SCP but was unable to identify deep intimal calcifications [61]. Sugiyama et al. [12] found that 12.7% of patients with ACS have 1 of 3 types of calcified plaques at the culprit site: SCP, eruptive CN, or CP. SCP were the most common aspect (67.4%), mainly located in the LAD and correlated positively with white thrombi. Compared to CN or CP groups, SCP was associated with the worst pre-PCI TIMI flow (*p* = 0.009) and the smallest minimum lumen diameter (0.63 ± 0.57 vs. 1.05 ± 0.82 vs. 0.92 ± 0.51 mm, *p* = 0.003). Furthermore, immediate post-PCI analysis [62] shows that SCP were associated with the smallest MSA (4.72 ± 1.37 vs. CN: 6.29 ± 2.41 vs. CP: 6.56 ± 1.13 mm^2^, *p* = 0.007) and the highest rate of periprocedural MI (60.3 vs. CN: 31.6%, *p* = 0.028). However, when comparing the long-term clinical outcomes of 258 patients among the 3 calcified plaques [63], the incidence of MACE (a composite of cardiac death, target-vessel MI, ischemia-driven revascularization) was lowest in the SCP group vs. CN and CP (10.1 vs. 32.1 vs. 13%, *p* = 0.001), mainly driven by the rate of target vessel MI (1.7 vs. 8.7 vs. 8.9%, *p* = 0.016) and ischemia-driven revascularization (5.6 vs. 13 vs. 16.1%, *p* = 0.029). Interestingly, layered plaque aspect was more frequently observed in the SCP group (*p* = 0.009), emphasizing its unstable nature as well as the possibility of rapid progression of this entity to a more severe stenosis by repeated thrombus formation and healing.

To date, no specific OCT cutoff values have been employed in terms of calcium depth, arc, or length to precisely define a SCP. Nevertheless, Zhan et al. [64] sought to investigate the minimum calcium depth that can predict plaque rupture. The study included 78 culprit lesions with superficial calcium divided into ruptured vs. non-ruptured caps. Mean calcification maximum arc and length were similar between the groups (83 vs. 76°, *p* = 0.94; 3.7 vs. 3.5 mm, *p* = 0.568). Calcium deposits were located more superficial in the ruptured than in the non-ruptured group (mean 50 vs. 110 µm, *p* < 0.001), as well as in MI compared to the unstable angina pectoris patients (mean 57 vs. 85 µm, *p* = 0.045). Interestingly, at a calcium depth ≤63 µm the lipid-rich calcified plaque was more prone to rupture (Se = 77.8%, Sp = 81.8%, AUC: 0.804, *p* < 0.0001). Ong et al. [32] proposed the 65 and 100 µm criteria to define superficial calcium deposits. The minimum calcium depth (*p* = 0.27) and the number of superficial deposits were similar between the STEMI and stable patient group (*p* = 0.35 using a 65 µm threshold; *p* = 0.84 using a 100 µm threshold).

Pinilla-Echeverri et al. [65] tested the efficacy of a super-high-pressure balloon (OPN-non-compliant) in the treatment of calcified coronary lesions. Half of the lesions (25 cases) were classified as SCP, 88% of which had a calcium score of 4. OPN-non-compliant was used in conjunction with other plaque modification techniques, especially cutting balloons (58%). Most lesions (92%) were treated with DES implantation and acceptable stent expansion (≥80%) was achieved in 40 cases (80%), while the remaining 20% achieved a reasonable MSA of 7.17 + 1.16 mm^2^. Calcium fractures were documented in almost all of the cases, with only one flow-limiting dissection and no other major complications.

End-stage renal disease represents a strong predictor of accelerated atherosclerosis, particularly at the coronary level. Using OCT, Chin et al. [66] compared 62 matched DM patients on hemodialysis to patients without chronic kidney disease and found greater calcium arcs, especially at the distal vessel segments, as well as a higher frequency of superficial thin (<0.5 mm) calcium (41.9 vs. 4.8%, *p* < 0.001) in the former group. The occurrence of such superficial calcium may lead to an overestimation of calcium burden using other modalities, such as IVUS or computed tomography. It is noteworthy that in another study [67], calcium burden and disposition was similar between DM and DM-end-stage renal disease patients, but calcium was more superficially located when compared to patients without DM or end-stage renal disease (0.14 ± 0.02 vs. 0.21 ± 0.02 mm, *p* = 0.01).

**Table 1:** Main OCT studies on SCP.

Study	Results
Matsumoto et al. [58]	First description of SCP
Saita et al. [61]	Histopathological validation of OCT for the identification of SCP
Sugiyama et al. [12]	SCP had the worst pre-PCI TIMI flow (*p* = 0.009) and smallest MLD (0.63 ± 0.57 vs. 1.05 ± 0.82 vs. 0.92 ± 0.51 mm, *p* = 0.003) vs. CN and CP
Nakajima et al. [62]	SCP had the smallest MSA (4.72 ± 1.37 vs. CN: 6.29 ± 2.41 vs. CP: 6.56 ± 1.13 mm^2^, *p* = 0.007) and the highest rate of periprocedural MI (60.3 vs. CN: 31.6%, *p* = 0.028)
Lei et al. [63]	SCP had lowest MACE rate (10.1 vs. 32.1 vs. 13%, *p* = 0.001) vs. CN and CP
Ong et al. [32]	Similar number of large (*p* = 0.27) and superficial calcifications (*p* = 0.35–65 µm threshold; *p* = 0.84–100 µm threshold) between STEMI and stable groups
Chin et al. [66]	Higher frequency of superficial thin calcium (41.9 vs. 4.8%, *p* < 0.001) when comparing dialysis vs. non-dialysis patients
Weber et al. [67]	Calcium was more superficial in DM or DM-dialysis group (0.14 ± 0.02 vs. 0.21 ± 0.02 mm, *p* = 0.01) vs. non-DM and non-dialysis group
Iwai et al. [68]	SCP had lower MACE (*p* = 0.0132) vs. CN

CN: eruptive calcified nodule; CP: calcified protrusion; DM: diabetes mellitus; MI: myocardial infarction; MACE: major adverse cardiovascular events; MLD: minimum lumen diameter; OCT: optical coherence tomography; PCI: percutaneous coronary intervention; SCP: superficial calcified plates; STEMI: ST-elevation myocardial infarction. TIMI: thrombolysis in myocardial infarction.

## 6. Eruptive Calcified Nodule and Calcified Protrusion

Due to increased mechanical stress calcified sheets may fracture leading to the formation of nodular calcifications that are associated with fibrin deposits [40]. These lesions may simply protrude into the lumen, overlayed by an intact and smooth intima surface (CP) or can penetrate the cap through the expulsion of small calcific nodules (eruptive CN) [12] (Figure 1). Intraluminal thrombosis has been demonstrated in both entities [12,62,63]. 

Jia et al. [69] provided the first in vivo OCT identification of calcified nodules, as the least common cause of coronary thrombosis in patients with ACS, while the same team further categorized culprit calcified plaques into three subtypes [12]. ACS patients with CN had worse post-stent parameters and clinical outcomes than those with plaque rupture or plaque erosion [70,71]. Compared to lesions without CN, lesions containing CN have a larger calcium burden (arc, length, and thickness) and a more superficial calcium localization [72]. Furthermore, in patients with ACS, CN are associated with more thrombi than in patients with stable CAD [72]. In a stable CAD cohort of heavily calcified lesions [73], the prevalence of CN was significantly higher in patients with device-oriented clinical endpoints than those without (41 vs. 18%, *p* = 0.002). Moreover, at a median follow-up of 2 years, the occurrence of CN together with medial dissection with calcified flap was associated with a higher incidence of device-oriented clinical endpoints (*p* < 0.001). Eruptive CN are more frequently observed in the ostial and mid-right coronary artery (40.5%), typically in areas with larger hinge movement [72]. When compared to SCP and CP, eruptive CN are associated with greater calcification index (median 3284.9 (IQR: 2113.3 to 5385.3) vs. 1644.3 (IQR: 1012.4 to 3058.7) vs. 472.5 (IQR: 176.7 to 865.2); *p* < 0.001) [12] and a higher frequency of stent edge dissection (47.4 vs. 17.5 vs. 20.0%; *p* = 0.032) and malapposition (94.7 vs. 58.7 vs. 0.0%, *p* < 0.001) on postprocedural OCT evaluation [62].

The prognostic impact of calcified plaque morphology using OCT has recently been investigated [63,68]. In the study by Iwai et al. [68] including 251 patients with moderate to severe calcification the rate of MACE-free survival (log-rank test, *p* = 0.01), MI (log-rank test, *p* = 0.01) as well as TLR (log-rank test, *p* = 0.04) was significantly worse in patients with CN compared to those with CP and SCP, respectively. Moreover, CN was an independent strong predictor of MACE in the multivariate analysis (HR 4.41, 95% CI 1.63–10.8, *p* = 0.004). In a sub-analysis of the CLIMA registry [74] the clinical relevance of CN or CP presence in non-culprit left anterior descending artery plaques was studied. At the 1-year follow-up, cardiac death and MI occurred in 20% of patients with CN vs. 2.7% in the CP group. Sato et al. [75] compared the post-PCI clinical outcomes of 236 patients with CN or CP at the culprit site. While MSA was similar between the groups (6.26 ± 2.26 vs. 6.18 mm^2^ ± 2.34 mm^2^, *p* = 0.71), stent expansion at the CN site was significantly greater in the CN group (89.2 ± 18.7 vs. 81.5% ± 18.9%, *p* = 0.003). A greater CN circumference, greater adjacent calcium arc and thickness and negative remodeling were associated with poor stent expansion. Interestingly, TLR drastically increased at approximately 6 months post-PCI in the CN group. At the 2-year follow-up CN were associated with a higher rate of TLR (18.3 vs. 9.6%, *p* = 0.04) and a trend toward more target lesion failure (19.8 vs. 12.5%, *p* = 0.11) compared to CP.

Isodono et al. [76] explored the role of eruptive CN development after stent implantation in 124 patients with in-stent restenosis (ISR). In ISR lesions with CN the rate of enlargement in the lumen and stent area tended to be greater that in ISR lesions without CN. Nevertheless, higher-pressure inflations and more frequent RA were used in the former group. Interestingly, the reduction in neointimal area tended to be smaller in the CN group. In a larger study including 651 ISR lesions [77], ISR with CN had an overall prevalence of 4.9% and was associated with calcified lesions (OR 12.441, *p* < 0.001), stent malapposition (OR 3.228, *p* = 0.005), hemodialysis (OR 3.633, *p* = 0.024), and female gender (OR 3.212, *p* = 0.036). At the mid-term follow-up, both ISR and TLR rates were higher in lesions with CN treated with either paclitaxel-coated balloon or DES compared to those without CN (43.8 vs. 25.0%, *p* = 0.023; 37.5 vs. 18.8%, *p* = 0.020). In a more recent study [78], in-stent CP alone or in association with CN was also found to be predictive of clinically driven TLR (HR 5.21, 95% CI 1.82 to 14.91, *p* = 0.002; HR 3.34, 95% CI 1.15 to 9.65, *p* = 0.03). In a high-risk dialysis cohort [79], 39% of patients had a CN at the culprit lesion before DES implantation. At the long-term follow-up, the CN group had a higher incidence of target lesion failure (a composite of cardiac death, target vessel MI, and clinically driven TLR) than the non-CN group (31.8 vs. 11.4%, *p* = 0.008). Furthermore, an in-stent CN was the cause of DES failure in the CN group in 73% of cases. The use of calcium modulation techniques such as intravascular lithotripsy seems to offer an answer to the poor results following PCI of lesions containing a CN or CP. In a multicentric prospective study [80] including calcified lesions, nodular calcification was found in 29.5% of lesions and following intravascular lithotripsy mean stent expansion at the nodule site was >100%. It is of note that dissection was more frequent than fracture at the nodule site.

## 7. Conclusions

Calcium is present throughout the whole spectrum of coronary atherosclerotic disease and can be accurately identified by means of OCT imaging, with the exception of cellular-level MC. Cellular-level MC seem to be ubiquitously found in fibrous caps, but only a small amount are related to plaque rupture. OCT-defined MC seem to be related to milder stenoses with more extensive inflammation and larger necrotic cores. SC are more frequently found in ACS patients, are associated with other plaque vulnerability features and can increase the risk of plaque rupture. Moreover, SC are predictive of adverse angiographical and clinical outcomes. Macrocalcifications may provide a plaque stabilizing effect but can lead to suboptimal angiographical results and increased MACE. Specific subtypes of macrocalcifications are related however to plaque vulnerability: SCP are the most common aspect and are associated with the smallest MSA and lowest MACE; eruptive CN are associated with the highest calcium burden and post-stent related complications and worse long-term clinical outcomes; CP are the least-frequently observed entity that may influence immediate post-stent results as well as long-term clinical outcomes.

## Data Availability

Not applicable.
